# Abstracts &c.

**Published:** 1889-06

**Authors:** 


### ABSTRACTS AND GLEANINGS.

---

**Chlorate of Potash in Epithelioma.**—M. Reclus has recently revived an old plan, somewhat in vogue forty years ago, of treating epithelioma of the skin by means of chlorate of potash, and is disposed to think that while this plan cannot be recommended wholesale as a substitute for excision, there are cases which, operative measures being, for one reason or another, inadvisable or impossible, may be satisfactorily treated in this way. One main point to be taken into consideration is the rapidity of growth. In order that chlorate of potash may have any chance of success, it must be employed for a considerable time. It is therefore only suitable in cases where the growth of the tumor is slow. Dr. Lemoine of Lille also has reported two cases of epithelioma, or cancroid, as he calls it, where chlorate of potash was employed with eminent success. In one case the tumor occurred in an old woman, occupying a large part of the left cheek, there being three enlarged glands at the angle of the jaw, and the skin around the ulcerated growth being tense, shining, and of a purple color. Half a drachm of chlorate of potash was given daily, compresses soaked in a solution being applied to the cheek, and a large pinch of the powdered salt being sprinkled over the surface of the tumor twice daily. The discharge of ichor, which had been abundant, soon began to diminish, and in about three weeks signs of improvement began to show themselves around the edges of the ulcer; in six weeks' time the diameter of the ulcer had diminished from 8 centim. to 4 centim., the surface having become dry, like that of a scirrhus, and the epidermis spreading over it a little more each day. The glands had become smaller; in eight weeks the surface had completely healed over. The internal administration was continued for a fortnight longer; since that time—some nine months before the report was made—no return of the signs of the disease had occurred. The second case was an epithelioma of the great toe, which had lasted about two years. This was treated by the internal administration of chlorate of potash and by its local application for about ten weeks, at the end of which time complete recovery is stated to have taken place. Unlike Dr. Lemoine, M. Reclus confines himself to the external application of the chlorate of potash. He finds that this is not suitable for cases where the growth affects the mucous membrane, because of the greater depth to which it generally penetrates under these circumstances. It acts best where the tumor is confined to the skin; but it may be employed where the junction of the mucous membrane with the skin is affected. —*Lancet.*

**Accidents Incidental to the use of the Exploring Needle.**—These may be divided into classes:

1. Those accidents resulting directly or indirectly from the traumatism produced by the needle.

2. Those cases of septic infection produced by the inoculation of healthy tissues during the withdrawal of the needle through them after introduction into more deeply seated infectious matter.

3. Those cases of septic infection produced by the introduction of needles not properly disinfected into healthy or diseased tissues or fluids.

I especially desire not to seem to exaggerate the dangers following the use of the exploring needle or to overestimate their importance. The following remarks, however, are suggested and seem to be justified by the cases reported :

1. The employment of the exploring needle is not infrequently attended by considerable danger, and a number of deaths have directly resulted from its use.

2. The indiscriminate, careless, and routine resort to exploration with a needle should not be resorted to without careful consideration of the conditions obtaining in each case and the results that may follow the puncture. The site for the puncture should be thoughtfully chosen, the puncture carefully made with complete antiseptic precautions, and the smallest needle that will answer the purpose employed.

3. The puncture of collections of fluids with tense walls in relation with serous surfaces should be, as far as possible, avoided, and, if it is resorted to, sufficient fluid should be withdrawn to relieve the tension upon the walls of the sac. In many cases certainly an exploratory operation would be attended by less danger.

4. In the introduction of the needle into deeply seated infectious matter the nature of the intervening tissue should be carefully considered.

5. The needle before use should be always thoroughly disinfected, preferably by heating in the flame of an alcohol lamp or a Bunsen burner.

6. The skin where the puncture is to be made should be rendered thoroughly aseptic by first scrubbing with soap and water and then washing with an antiseptic solution.

7. The dangers attending the use of this valuable adjuvant in diagnosis should not in the slightest interfere with its employment in properly selected cases, where the due precautions are observed as to its use.—*The Epitome.*

**The Hypodermic use of Ergot in Facial Neuralgia.**—Severe facial neuralgia is an affliction which calls out the deepest sympathy of the physician, and tests to the utmost his therapeutic resources. Any method of treatment which offers even the slightest chance of relief, is gladly welcomed in chronic and obstinate cases. In the *Peoria Med. Monthly* of January, 1889, Dr. Steward states that, for the relief of facial neuralgia, ergot injected beneath the skin with a hypodermic syringe is incomparably superior to either aconite or gelsemium. He has used it in this way for six years, and has failed to give relief in but one case, in which case there was organic disease. Generally, one injection relieves the pain permanently. Sometimes two injections are required, and in a very severe and obstinate case which had baffled several physicians, he used three, with complete cure of the disease. He puts it into the temple, as nearly over the seat of pain as convenient. He uses the plain extract, which he has made so that one minim represents two grains of ergot, throwing in eight to twelve minims, blood-warm, at one injection, without diluting. The extract must be from pure and good ergot, and must be reasonably fresh. If kept long, it becomes inefficient and irritating. When properly prepared and fresh, it produces more or less pain for ten or fifteen minutes, and when this pain subsides the neuralgia has usually disappeared, not to return. In sciatica and other forms of neuralgia he has used the same method without decided benefit.—*Maryland Medical Journal.*

**The Medicinal Treatment of Intestinal Obstruction,**—Hofrath Professor Nothangle recently made a notable contribution to the discussion of the pathology of ileus and of its treatment by other measures than laparotomy, at a meeting of the *medicinisches Doktoren-Collegium* of Vienna, a stenographic report of which was published in the *Internationale klinische Rundschau* for March 17th. So far as the pathology was concerned, his remarks related chiefly to the phenomena observed when artificial intestinal stenosis was established by means of a ligature in experiments upon the lower animals. In such experiments the animal is kept anesthetized for a number of hours, with the body immersed in a blood-warm half per cent. solution of common salt. The effect of the ligature upon peristalsis varies according to whether it is applied to a quiescent portion of intestine or to one in which a peristaltic wave is already in progress. In the former case it does not give rise to tempestuous peristalsis; in the latter it does. When the ligature is tight enough to cause effectual obstruction, violent peristalsis sets in in the portion of intestine above the constriction, the contents are forced down against the stricture, and the intestine just above the ligature becomes over-distended and at length paretic; but no antiperistalsis is observed. Fecal vomiting is accounted for by the fact that it is in the direction of the stomach that the least resistance is encountered. The intense pressure at the seat of stricture, brought on by the stormy peristaltic action set up above it, gives rise not only to paresis and distension, but also to ulceration or gangrene in some cases; and, independently of either of these occurrences, collapse and death may take place as the direct consequence of intense irritation of the nerves, especially of the splanchnic nerve.

As regards the treatment, therefore, every indication points to measures capable of soothing the irritation and calming the intensified peristaltic action. Above all things purgatives should be avoided. In cases of simple fecal accumulation they may be used with advantage, but they are worse than useless in cases of mechanical obstruction. No food of any sort should be taken into the stomach, but the patient may be allowed to take a little liquid food into the mouth occasionally and then reject it. Although peristalsis above the stricture is to be restrained, it may be encouraged below that point without harm and sometimes with benefit; occasionally it seems to relax the constriction and admit of the passage of some fecal matter. This is best accomplished by employing enemata of warm salt and water. Opium should be given in large doses, preferably in the form of the crude drug, but if that is not well borne by the stomach, morphine may be used subcutaneously. Opium not only restrains peristalsis, but tends to sustain the patient's vital power. Nothangle thinks that Kussmaul's method of treatment by washing out the stomach has been overestimated. He thinks that most of the successful cases reported have not been well marked, and that the unsuccessful ones have been largely witheld from publication. Little more value is attached to the use of large enemata, electricity, belladonna, nicotine or mercury. —*N. Y. Med. Jour.*

**Crude Petroleum as a Surgical Dressing.**—While I was sojourning on a ranch in Iowa this summer, a cowboy, while driving a large herd of cattle, was crowded against a barbed wire fence, inflicting three lacerated wounds. One, about seven inches long, commenced about three inches below the knee and running backward and downward denuded the fibula.

My professional services being required, and, not having the necessary appliances at hand, I cleansed the wound and daubed it with crude petroleum and brought the edges together with a roller bandage, leaving a small opening at the dependent part for the expected suppurated drainage.

I expected as a result of the injury either suppuration, erysipelas or tetanus. As the patient was in great pain I injected a quarter of a grain of sulphate of morphine. Next morning he was free from pain. I examined the wound daily, but was agreeably surprised to find neither fetor nor discharge of pus. The dressing was allowed to remain undisturbed for a week and upon its removal the wound was found perfectly healed with the exception of a single place. I used adhesive strips to bring this together and in eleven days from the date of injury the patient was up and around. Cold water applications were used frequently for several days after the receipt of the injury. In the light of this experience I regard this remedy as a valuable adjuvant in such cases of surgery.—*Dr. McMurray in St. Louis Medical Journal.*

**Action of Iodide and Bromide of Potassium upon Morphine.**—Dr. H. Kunz, after numerous experiments, performed for the purpose of determining the identity of the hydriodate and hydrobromate of morphine, has arrived at the following conclusions :

1. It is necessary, as well as possible, to avoid using in prescriptions the iodide and bromide of potassium in combination with a salt of morphine ; or when they are so used to prevent, by the addition of an alcoholic liquid, the formation of a precipitate.

2. All prescriptions containing these salts ought always be labelled "shake before taking-"—*Journal de Medicine.*

**The Microbe as a Factor in Disease.**—The prevailing opinion now seems to be that the microbe is not the all-important factor in disease, but that the substances elaborated by it do the harm. It is certain that in some cultivations the bodies evolved cause a cessation of the growth of the microbe, and this fact is now taken as a starting point for a new theory of inoculation against disease. Professor Bouchard has said in his lectures that there ought to be careful separation of the soluble matters secreted by the micro-organism from the toxic substances which might exist in connection with them. The former only were suitable for inoculation. It is possible, he thought, that they might come into use for internal medication to arrest the progress of a disease which had already begun, rather than for inoculation. Thus it might be that the substances that arrest the development of micro-organisms in cultivations could be used to combat their growth in the body, and thus to put an end to the disease the micro-organism had caused. Professor Peter said that he never had believed that the microbes themselves did any harm, but that their presence gave rise to the formation of poisonous alkaloids. Professor Bouchard was of the opinion that inoculated matter was not retained within the tissues to act as certain substances did which were put into cultivation to prevent the growth of micro-organisms, but that it modified cell-nutrition to such an extent that the cells became permanently, or at least for a long period, incapable of being again affected by the substance which had modified them originally. The inoculations practiced by him of the urine of animals suffering from various diseases had appeared to confer immunity from the disease affecting the animal from which the urine had been taken. This would seem to indicate that a modification of the virus, similar to the attenuation produced artificially, as in the case of anthrax, had occurred spontaneously in the system.—*Paris Letter*, *N. Y. Med. Jour.*, March 2d, 1889.

The prognosis of locomotor ataxia is generally bad. A new treatment has been suggested, which, according to report, bids fair to change the future of tabetic patients. We believe that the new treatment was first suggested by Dr. Motchoukowsky, of Odessa. The treatment consists of suspension by means of Sayre's suspension apparatus.

"Three minutes may be regarded as the proper average time" of suspension. This should be done every other day. "The results obtained were especially remarkable in the cases of progressive locomotor ataxia," in the later stages. "Prof. Charcot has obtained the most encouraging ameliorations in a class of diseases which seem thus far to have defied all the efforts of therapeutics." (Med. Abstract.)

**Phenacetine** is charged with producing a condition of alarming intoxication in a patient aged thirty-eight, suffering from migraine. Two doses of fifteen grains each had been taken at short intervals.—*Med. Chips.*

**Chloroform in Dyspepsia.**—We copy from the *Medical News* several formulas by different authorities for giving chloroform in dyspepsia:

Chloroform administered in the various forms of dyspepsia overcomes fermentation and flatulence; it is best given in the following formulas:

1. Method of Dr. Wils: From ten to twenty drops of chloroform, to be taken in a few spoonfuls of sweetened water, in flatulent dyspepsia. After a few minutes eructations occur, followed by improvement.

2. Method of Dr. Huchard: Administer before each meal one dessertspoonful of the following:

| | | | |
|---|---|---|---|
| Chloroform water, | 150 | parts. | |
| Mint water, | 30 | " | |
| Water, | 120 | " | M. |

Or from eight to ten drops of the following mixture in a wineglass of water:

| | | |
|---|---|---|
| Tincture of nux vomica, | äā ℥j | |
| Tincture of gentian, | | |
| Tincture of anise, | | |
| Chloroform, . . . . | gtt. xx–xl. | M |

An appropriate diet and oxygenated waters at meal times form part of this treatment.

3. Methods of Dr. Regnault and Laseque: This treatment applies particularly to painful dyspepsias with dilatation of the stomach:

| | | | |
|---|---|---|---|
| Chloroform, . . . | 150 | parts. | |
| Orange-flower water, | 50 | " | |
| Water, . . . . . . | 100 | " | M. |

One dessertspoonful to be taken at intervals of fifteen minutes until the pain ceases.

Or the following for the same affections.

| | | |
|---|---|---|
| Chloroform water, | 150 | parts. |
| Tincture of anise, | 5 | " |
| Water, . . . | 145 | " |

Chloroform has long been used internally in Louisville in the treatment of dyspepsia. It has been found useful in vomiting both in Asiatic cholera and cholera morbus. But it is a drug to be used with some caution. Twenty drops of chloroform is a large dose, occasionally large enough to produce very unpleasant symptoms. The *American Practitioner* contained, now many years back, the report of a case wherein ten drops, taken in sweetened water, caused dizziness, faintness and partial loss of consciousness, phenomena which persisted several minutes. This particular instance may, it is true, have been one of idiosyncracy, for ten drops of chloroform would seem really not to be a great quantity to give at one time; yet it is well to have in mind the fact to which we here call attention.—*American Practitioner and News.*

**Cancer Treated by Creosote.**—The treatment of cancer is naturally much like that of phthisis. Creosote, which has been found to stimulate the nuturation of the blood corpuscles, is of equal value in cancer and in phthisis. He has obtained decided results in the treatment of cancer with the following preparation :

℞ Creosoti puri.
Sodii bicarb.
Olei morrhuæ, aa f. ℥ v.

M. Put in 100 gelatine capsules. Take three capsules three times daily, after each meal.

A very eligible substitute for creosote is creolin, as it is not only cheaper than the former, but is also a stimulant of digestion. He prescribes :

℞ Creolin, m xv.
Ext. glycyrrhizæ, q. s. ut ft. pill. No. 100.

Sig.: Three pills, three times daily.

Locally, Dr. Neudorfer prescribes with the above pills.

℞ Creolin.
Ichthyol.
Iodide of potash, aa gr. xjj.
Vaselin.
Lanolin, aa gr. xv.

M. F. ung. Sig.: Rub into the part three times daily.

Neudorfer boasts of three actual "cures" of cancer obtained with this treatment.—*Medical Waif.*

**Treatment of Typhlitic Diseases.**—Dr. A. M. Jacobus, of New York, in an interesting study of these affections (*Med. Record*, Feb. 2d. 1889), arrives at the following conclusions :

1. That typhlitic diseases are intra-peritoneal, that in nearly every instance they originate in inflammation or perforation of the appendix or cæcum, usually caused by fæcal impaction or foreign bodies, that even though these diseases frequently do not primarily end in suppuration, still their tendency is to recur and eventually end fatally by septicæmia, or shock from ruptured abscess, or purulent peritonitis.

2. That in view of the foregoing, all diseases of this region, giving symptoms of inflammation or peritonitis, without regard to the question of pus or abscess, and which do not yield promptly to the modern medicinal treatment and are progressive, should be treated by laparotomy at the earliest period possible.

That when peritoneal or intestinal adhesions or pus sacs exist, they should always be broken up, the abdominal cavity thoroughly douched with sterilized plain water, at a temperature of 105° F., the wounds sutured, and the cavity drained by glass tubes.

4. That, excepting in undoubted cases of simple post-cæcal abscess unaccompanied by peritonitis, the incision should be made either vertically over the caput coli (after Sands), or in the linia alba, below the umbilicus, and preferably the latter operation when extensive peritonitis or adhesions are suspected.

5. That, whether in operative cases or not, opiates should be used as little as possible; but, on the other hand, saline or vegetable cathartics (followed by enemata) should be used from time to time to relieve the inflammation, tympanites, pain, and fever, and particularly to drain the peritoneal cavity of serous or purulent effusion.—*College and Clinical Record.*

**Headache.**—We would note the following as being specially valuable:

"Note on the Relief of Migranous Headache," by Dr. Jas. Little. The author has found that in this persistent trouble, which is becoming so common among American brain workers, he has obtained the best results from twenty grains of salicylate of sodium given in a wineglass of water, made effervescing by the addition of a dessertspoonful of citrate of caffein, immediately on rising in the morning, and repeated if necessary. He also advises the use of the following pill:

| | | |
|---|---|---|
| ℞ | Arseniate of Sodium, . . . . . . | gr. $\frac{1}{12}$ |
| | Ext. Cannabis Indica, . . . . . | gr. $\frac{1}{6}$ |
| | Extract Belladonna, . . . . . . | gr. $\frac{1}{3}$ |
| | Valerianate of Zlnc, . . . . . . | gr. ij |

M. Sig.: To be taken after breakfast and dinner.

**The Local Treatment of Diphtheria by Antipyrine.**—Barato Ribeiro claims success from the local treatment of diphtheria by antipyrine, the throat being thoroughly swabbed with a ten per cent. solution. Each swabbing was preceded by an application of cocaine. A solution of the same strength was also used frequently as a gargle. In nasal diphtheria this Italian writer counsels irrigations with the same solution, and insufflations of antipyrine finely pulverized. Internally antipyrine was given with good effect in combination with port wine and water, two grains every two hours.

Ribeiro regards antipyrine as a safe and efficient antiseptic and germicide in diphtheria.—*Ib.*

**Hydrate of Chloral in Chapped Nipple.**—In the *Journal Akusherstva i Jenskikh Boleznei*, Dec., 1888, p. 892, Dr. Ivan A. Mitropolsky, of Moscow, highly recommends chloral as an excellent local means for fissured and excoriated nipples. The latter should be kept covered with compresses (soft linen) soaked in a solution of half a drachm of chloral in three ounces of water. The compresses should be changed every two and a half or three hours. When a prolonged application is necessary, it is advisable to use a weaker lotion (½ dr. to 6 oz). The solution leaves a thin, whitish, firmly adherent film over the diseased surface, which does not disappear by suckling. Pain and tenderness are said to be strikingly relieved almost immediately; the lesions rapidly healing. The chloral compresses do not produce any bad effects on nurslings.—*St. Louis Medical and Surgical Journal.*

**Sweet Spirits of Nitre a Solvent of Salicylic Acid.**—As the administration of salicylic acid has become so extensive, and as a good solvent is desirable, I wish to make known through the Age that spiritus nitrici dulcis (sweet spirits of nitre) is the best solvent. I have been prescribing it nearly four years in the treatment of malarial fever with uniform success, without the use of quinia. I employ this formula:

℞ Salicylic acid, ʒj.

Sweet spts. nitre, ℥ iv.

Mix. Sig.: One teaspoonful every two hours for children; two to four teaspoonfuls for adults.

It can be diluted with water if necessary, and can be combined with veratrum, gelsemium, aconite, etc. One ounce of nitre will dissolve sixteen grains of the pure acid and makes a clear solution. If that quantity of acid is increased, on the addition of water the acid is precipitated; the mixture becomes semi-solid. Add a little more of the nitre and it becomes a clear solution again. I will add that in the treatment of intermittent and remittent fevers salicylic acid is a valuable remedy, generally relieving those diseases within forty-eight hours.—*Medical Age.*

**Exalgine, the New Analgesic.**—Exalgine is the name given to a new derivative of the aromatic series, orthomethyl-acetanilid, discovered by Brigonnet, of the Cochin Hospital, and which has suddenly leaped into extraordinary favor as an analgesic in France. The name (*ex*, privative, and *algos*, pain) is significant of its qualities. The formula is $C_9H_{11}NO$ (or $C_6H_5.C_2H_3O.NCH_3$), and the substance is one of the three isomeric (para, meta, and ortho) methyl derivatives of acetanilid. It occurs either in fine acicular or long tablet-like crystals, accordingly as it is obtained by evaporation from solution, or by fusion thereafter. It is sparingly soluble in cold water, more soluble in hot water, and extremely soluble in very dilute alcohol, or in water slightly alcoholated. Physiologically it acts very much like analgesine, having, however, more effect upon the sensory and less upon the thermogenic centers than this substance. Its therapeutic effects are obtained in doses of from 4 to 6 grains, administered at once, or from 6 to 12 grains taken in two doses in the course of twenty-four hours, and are powerfully analgesic, subduing the element of pain in all forms of neuralgia, including visceral. Like all new remedies of this sort, it is at present on its good behavior, as it were, and it is claimed by MM. Dujardin-Beaumetz and G. Bardet that it has in their hands up to the present exhibited no evil sequelæ, being free from the rash, cyanosis, etc., so frequently observed after the ingestion of antipyrin and acetanilid. Exalgine is eliminated by the urine, upon the quantity of which it exercises a marked effect, acting like the antipyretics of the same group, diminishing the quantity of the secretion. In diabetes it also diminishes the quantity of sugar eliminated. Like all of the derivatives of the aromatic series, it is antiseptic and antithermic, as well as analgesic, and possesses the latter quality in a comparatively superlative degree, being more efficient, in doses less than half so great, than antipyrin. The following are the formulæ for its exhibition, as given by M. G. Bardet in *Les Nouveaux Remedes* for March 24:

1. *Antineuralgic potion* of Exalgine.

℞ Exalgine, . . . . . . . . . ʒi

Kirschwasser, . . . . . . . . ʒx

Simple syrup, . . . . . . . ℥i

Distilled water, sufficient to make . . . ℥v

Dissolve the exalgine in the kirsch water and add the syrup and water. The dose is from 1 to 3 tablespoonfuls during the course of of the day.

2. *Solution* of Exalgine.

℞ Exalgine, . . . . . . . . . ʒi

Rum, . . . . . . . . . . ʒx

Distilled water, sufficient to make . . . ℥v

Proceed as before. The dose is the same as above.—*St. Louis Med. and Surg. Journal.*

**The Disinfection of Surgical Instruments.**—Dr. L. Freeman, in a paper recently read before the Cincinnati Academy of Medicine (*Cinn. Lancet-Critic*, March 2d, 1889), states that upon the thorough disinfection of our fingers depends so much that every physician should be familiar with the very best process for accomplishing this end. Furbinger has furnished us with perhaps the most reliable and convenient method. It is as follows:

1. Clean the nails well with a knife.
2. Brush the hands for one minute with warm soap and water, giving special care to the nails.
3. Wash and soak the hands for one minute in alcohol over 80%, to remove fatty matters and assist disinfection.
4. Then immediately into a three % solution of carbolic acid, washing and rubbing the hands hands carefully for a minute, not forgetting the nails.

When this process has been carefully gone through with, the surgeon can be reasonably certain that hands are aseptic. His difficult duty is then to keep them so; in other words, to avoid touching all unsterilized objects.

To recapitulate:

1. Micro-organisms of all kinds swarm about us; some are pathogenic, the majority harmless, so that a wound may or may not become affected, as chance directs.
2. Not enough attention is paid by most surgeons to the disinfection of their instruments. An examination of a series of instruments revealed a large number of various kinds of bacteria. No pus-formers were found; but this must be regarded as accidental, as the conditions were favorable for their existence.
3. The disinfecting agents now in use, carbolic acid and corrosive sublimate, are not to be depended upon, as shown by the experiments of others and by my own observations.

4. The best way to disinfect Instruments is to expose them for five minutes to streaming steam at 100° C., or boil them for the same length of time in a closed vessel, both before and after an operation.

5. As far as possible, only smooth, seamless instruments should be used.

6. Instrument cases should be looked after as presenting a source of infection.

7. The hands should be carefully cleaned with soap and water, soaked in alcohol, and then washed in an antiseptic solution, each step of the process to occupy one minute.—*Col. and Clin. Rec.*

**Reflex Neuralgia Caused by Tapeworm.**—At a recent meeting of the St. Petersburg Society of Naval Medical Men, Dr. Sadovsky related the following curious case (*Meditzinskia Pribavlenia k'Morskomu Sborniku*, January, 1889, p. 66): A stout, healthy gentleman, aged 56, sought the author's advice on account of an agonizing facial neuralgia of about six years' standing. The paroxysms occurring in the region of the inferior branch of the left trigeminus had been coming most frequently, especially during daytime, free intervals lasting but a few minutes. To alleviate his agony, the patient had been in the habit of rubbing the painful region with his very long and thick beard, which procedure, with time, had given rise to the formation of a corn, as large as a shilling, situated on the lower lip, close to the left angle of the mouth. Persistent treatment by all possible means, including electricity, injections of osmic acid, hypnotic suggestions, etc., had utterly failed to bring any relief to his sufferings. Dr. Sadovsky's inquiry elucidated the fact that the gentleman had been suffering from tapeworm for many years. Starting from the supposition that the neuralgia might be caused, after all, by a gastro-intestinal irritation kept up by the parasite, the author administered ethereal extract of male fern (3 grammes) with kamala (5 grm.) and on the next day a full dose of castor oil. An enormous mass of tænias was expelled in the course of the three succeeding days. The neuralgic pains ceased as if by magic and never returned (three months elapsed since his cure),—*Ib.*

**Treatment of Insomnia.**—Dr. W. H. Thompson, of the New York Hospital, (*Medical and Surgical Reporter*), prescribes as follows in insomnia due to worry or mental strain. This variety he considers most dangerous, as it may lead to epilepsy or insanity.

| | |
|---|---|
| ℞ Acid hydrocyanic dil., . . . . . | gtt xvi |
| Sol. morphin. Magendies, . . . . . | ʒj |
| Chloral. hydrat, . . . . . . . . | ʒjss |
| Syr. zingiberis, . . . . . . . | ℥j |
| Aquæ camphoræ, ad . . . . . . . | ℥vi |

M. Fiat Sol.

Sig.: Dose, for ordinary case, a tablespoonful ; for severe case, two tablespoonfuls.

This prescription is also good for the sleeplessness of fatigue,—*Ib*,

**A new remedy for Cholera.**—Loewanthal (*Acad. des Sciences*, Session December, 1888) has concluded a course of experiments undertaken to find an antidote to the virus of cholera. This toxic principle is now, according to the newest pathology, regarded as the product of Koch's cholera bacilius,—a ptomaine, in fact, which is destroyed by cultivation in artificial nutrient media. Loewenthal has found that a pure culture of these cholera bacilli in peptonized broth, previously sterilized, is absolutely inoffensive to animals—as white mice—naturally susceptible to poison; the bacilli ceasing to produce the noxious ptomaine.

The first aim of Loewenthal's experiments was to render to the cholera bacillus, by a process of the laboratory, the toxic property which it possesses when fresh, but which is lost on cultivation. After many fruitless essays, he believes that he has succeeded with a paste which contains pancreatin, and the composition of which is as follows: fresh pork (muscle), hashed, 500 grammes; pancreas of hog, hashed, 200 grammes; bean flour, 100 grammes; peptone, 15 grammes; grape sugar, 10 grammes; common salt, 5 grammes. These substances, mixed with water or milk, give a soft paste, almost liquid, which is rendered alkaline by a little potash, and then steralized by hot steam. The cholera bacilli, which by culture have lost their pathogenic properties, are allowed to breed in this artificial paste. They immediately secrete their virulent ptomaine, which, when inoculated in mice, either kill these animals or make them intensely sick.

By varying the elements of his culture mixture, Lowenthal finally satisfied himself that it is the pancreatic juice which, in presence of albuminoid and peptonized substances, determines the pathogenic or poison-secreting action of the bacillus. All the other culture media (peptone-gelatine, agar-agar, bouillon) assure the development of the bacillus, but no toxic matter is produced.

This peculiar action of the pancreatic juice being understood, we have, says Lowenthal, an explanation of the phenomena of cholera in man. The bacilli, after being ingested, escape the stomach, and entering the intestine, produce there, with the help of the pancreatic juice, the same toxic matter which is produced in the pancreatic paste, the latter being a coarse imitation of the contents of the duodenum; this toxic matter is absorbed and the restoration or death of the patient depends on the quantity of poison absorbed and the resistance of the organism. This experimental fact is in harmony with the anatomo-pathological fact that the bacilli of cholera remain always confined to the intestine, as well as with the "fulminant cases," and the experiments of Nicoti and Reitsch, and those of Koch on animals.

This point being once determined, Loewenthal asked himself if there might not be some substance inoffensive to man which, introduced medicinally, would prevent the development of the cholera poison in the intestine. To determine this, he first experimented with his pancreatic paste, trying various antiseptic agents which he thought might prevent the active functional operations of the bacilli and the genesis of the toxic ptomaine. Any agent, he reasoned, which can accomplish this *out of the body*, might be relied upon to do the same *within the body*, and thus become a specific (preventive and curative) remedy for cholera.

This remedy Dr. Loewenthal announces that he has found in salol, the salicylate of phenol, discovered in 1886 by Nencki, of Berne. This powerful antiseptic is decomposed in the organism by the pancreatic juice, the same agent which renders toxic the cultures of the cholera bacillus in the pancreatic paste. A multitude of experiments have assured him that salol in presence of fresh pancreatic juice is invariably fatal to the cholera baccilli in his laboratory culture-tubes; and he has determined the quantity which is sure to effiectually sterilize his cultures, namely, two grammes of salol to every ten grammes of the paste; a smaller dose, however (as ten centigrammes), renders the bacilli inactive.

It is known that salol can be taken in pretty large doses (as much as ten to fifteen grammes a day) by man with comparative impunity.

It must be added that the above interesting laboratory experiments, conclusive as they seem to be to their author, who has full faith that he has found a sure specific for cholera, still lack clinical confirmation which comes from a series of carefully conducted experiments on animals.—*Boston Med. avd Surg. Jour.*

**Medicinal treatment of Menstrual Disorders.**—The management of such diseases without physical examination is often demanded of the physician in the case of young girls, or others who will not submit to local treatment until driven to it by long continued suffering. The author's experience is given in the following resume: *Ammenorrhœa.* General tonics, iron, arsenic, cod liver oil. The salts of manganese, particularly the binoxide, have given the most satisfactory results. The permanganate of potassium often gives excellent results, but like all compounds of manganese, except the binoxide and the lactate, causes too much gastric irritation to be of great value.

*Dymenorrhœa.* The author has had best results from three to five drop doses of tincture of pulsatilla, cannabis Indica, viburnum, camphor, belladonna and antipyrin. Binoxide of manganese has also done good service in doses of six grains *per diem.* Heat, as applied by douch or sitz bath, is a valuable adjuvant.

*Menorrhagia.* In this condition the most reliable remedies are ergot, hydrastis, digitalis, sulphuric acid, fluid extract of gossypium, and gallic acid.

The author gives a review of the opinions of many prominent gynæcologists on these subjects, and finds much lack of unanimity as to certain drugs, and finds that the number of remedies which are vaunted as useful in the treatment of any particular form of menstrual disorder is usually in inverse proportion to its amenability to treatment. —*Editorial in Medical Record.*

**Carbolic Acid Inhalations in Whooping Cough.**—The use of carbolic acid inhalations give, it is claimed by Pick and others, decidedly beneficial results in whooping cough. It is to be used either pure or diluted with an equal quantity of water. Inhalations may be given hourly for ten minutes at a time. The reports of its use show no symptoms of carbolic acid poisoning, and the paroxysms are lessened in severity and frequency. It can be used with an ordinary chloroform inhaler, or with a large pasteboard tube, containing absorbent cotton moist with the solution, and the ends of the tube closed with coarse cheese cloth or gauze.—*Indiana Med. Journal.*

**Permanganate of Potassium in Rattlesnake Bites.**—There has been much written of late years upon the efficiency of permanganate of potassium in snake bites. Dr. J. W. Carhart writes to the *Texas Courier Record of Medicine* that he has treated several cases of rattlesnake bites by the hypodermic injection of permanganate of potassium with invariable success. He injects a solution of five grains of the salt to an ounce of water near the site of the bite and applies poultices to the swollen part. He does not use any alcoholic stimulants at all in connection with this treatment.—*St. Louis Medical Journal.*

**Bloodless treatment of Ingrowing Nail.**—Dr. Patin recommends the following procedure for removal of ingrowing toe-nail, which he has employed with excellent results in all his cases. After thorough cleansing of the nail, a solution of gutta percha, 10 parts in 80 of chloroform, as applied with a brush to the interstices between the nail and the granulations. This is repeated several times on the first day, and subsequently at longer intervals. By exercise of care and patience it will be found that the nail is gradually lifted from the underlying parts, and can then be removed without pain with the scissors. If a properly fitting shoe is worn, no recurrence need be apprehended. The solution applied in this manner exerts a double effect—the chloroform is anæsthetic, and the gutta percha acts mechanically, forcing its way between the granulations and the nail, and finally liberating it from its abnormal position.—*Gaz. des Hospiteaux.*

M. Pasteur recently presented to the Academie des Sciences, Paris, a communication, the work of MM. Roux and Cumberland, claiming that immunity may be gained against septicæmia by the inoculation of certain soluble substances. It is stated that the septic microbe, in its growth, develops chemical products which react upon itself, until finally it is destroyed by its self-engendered poison.

Roux and Cumberland have isolated these soluble products, and, by injecting them into guinea-pigs, have made these animals incapable of being affected by the septic vibrio.

These experiments have not been applied yet to the human subject; but if they should prove successful, their importance would be very great in the prevention of blood-poisoning by absorption of putrescent products.—*Popular Science News.*

**A Useful Formula in Skin Disease.**—I have been so successful in treating certain cases of skin diseases, that I thought I would write a communication on the subject. One case, that of a printer, who was affected with a very bad eczema of a chronic nature, on both hands (dorsal aspect), presented himself. His hands were so sore and inflamed that he could not work at his trade for weeks at a time. Hypertrophy of the skin, large scabs with cracks between, from which issued pus and other discharges, were the conditions as near as I can describe. I prescribed the following mixture with instructions for him to apply three times a day, very thoroughly at night, at the same time keeping the parts protected by cloth gloves.

℞ Ac. salicylic, ʒij.

Ac. boracic, ʒiss.

Biborate soda, ʒij.

Alcohol.

Glycerin, ää q. s. ad ℥iij.

M et ft. lotion

There was immediate improvement, and by sticking to the above treatment for three months his hands are now comparatively well. I was myself affected with a very troublesome hyperidrosis, or hypertrophy of the sweat glands of the palms of both hands, and bromodrosis of both feet. I used the same remedy in a similar manner and I was surprised to see what good results followed. It is an excellent remedy in many skin affections, as acne, etc. It will pay any man who has an obstinate case of skin disease of a non-specific nature to give it a trial. I generally incorporate about ten drops of ol. bergamont with the formula to give it an agreeable smell.—*Dr. Frank M. Scott in Medical Age.*

**For Rigid Os and Perineum.**—Dr. Abraham Livezey, of Yardley, Pa., for a great many years has used a rectal injection of a teaspoonful of tincture of lobelia in a sufficient quantity of mucilage, immediately after a pain. This evacuates the bowels, making the labor more cleanly, and relaxes the os and perineum, rendering the use of forceps unnecessary in most cases. The well.known combination of tincture of gelsemium and lobelia in suitable doses by mouth will act almost as promptly.—*Med. World.*

**To Remove Hiccough.**—Dr. Bottrich has found the best proceeding, to take a very deep inspiration and hold the breath as long as possible. If the breath be kept just past a rising singultus the latter generally ceases.—*Med. Brief.*

**Chamomilla.**—Dr. Ringer, in "Hand-Book of Therapeutics," says: "In the ordinary summer diarrhœa of children, often occurring during teething, characterized by green vari-colored and slimy stools, the infusion of small doses often proves very useful, especially when given at the commencement."—*Ib.*

**Prostatic Enlargement.**—Importance is attached to a new operation for the relief of this condition, devised by Dr. Hunter McGuire, of Richmond. The operation is similar to supra-pubic cystotomy for stone. The only difference is that he made the opening into the bladder as low down on its anterior wall as possible, and left the opening in the skin at the upper end of the incision. A drainage tube was kept in for a short time. The result was that the patient passed his urine through the artificial urethra thus formed. The artificial urethra did not leak, nor did the urine dribble away, no matter what the position of the man's body was. The urine was retained for several hours, often from four to six, and then passed in a strong stream thrown several feet from the body, the last coming in jets as from a natural outlet. The improvement in the patient upon whom he had done this operation had been wonderful. The artificial urethra or fistula had the same relation to the bladder that the spout of a coffee pot has to the pot.—*Clin Record.*

**What Prohibition does.**—A gentlemen who has spent several months in Kansas says: " Kansas boys ten years old and under never saw a saloon since they can remember. They never saw a man under the influence of liquor. On arriving at man's estate, they will have no more desire for drink than they will have for opium or hasheesh." This is very true, for we believe that, in very many cases, the habit of drinking liquor has found its origin in the imitative propensities of humanity; and where the example is not set, the habit will not be acquired.—*Annals of Hygiene.*

**Acetanilide as an External Sedative.**—I have not noticed any reference to antifebrin as a local sedative or antipyretic, and am unaware of its having been used as such. It occurred to me to try the effects of it in this way, and the remarkable results in the few cases in which I have prescribed it leads me to anticipate that a more extended use of this remedy as an external application will be found of great value. Country practice, especially in such a healthy district as this is, does not afford much scope for experience in skin diseases, and I have only tried it for a few months. My usual plan is to prescribe it with lanolin or vaseline in the proportion of twenty grains to the ounce, combined with other ingredients that seem applicable to special cases. In obstinate irritable ulcers it soothes the pain and subdues the inflammation. In psoriasis, combined with some mercurial preparation, it acts like a charm. I have also tried it in erythema, erysipelas, eczema, herpes, urticaria, and other complaints associated with considerable irritation, and have found it a most useful adjunct to suitable remedies.—*A. H. Newth, M. D., in the Lancet, April 6, 1889.*

**Gelsemium.**—For cold in the head, while in the acute congestive stage, no better remedy. One good large dose, say ten minims of the fluid extract, taken upon retiring at night, will effectually dispose of this troublesome and uncomfortable affection. One dose is usually sufficient.

